# Canagliflozin, serum magnesium and cardiovascular outcomes—Analysis from the CANVAS Program

**DOI:** 10.1002/edm2.247

**Published:** 2021-03-13

**Authors:** Katherine M. Wang, JingWei Li, Vivek Bhalla, Meg J. Jardine, Bruce Neal, Dick de Zeeuw, Greg Fulcher, Vlado Perkovic, Kenneth W. Mahaffey, Tara I. Chang

**Affiliations:** ^1^ Division of Nephrology Stanford University School of Medicine Stanford CA USA; ^2^ The George Institute for Global Health University of New South Wales Sydney NSW Australia; ^3^ Department of Cardiology Xinqiao Hospital Army Military Medical University Chongqing China; ^4^ Department of Renal Medicine Concord Repatriation General Hospital Sydney NSW Australia; ^5^ Kidney Health Research NHMRC Clinical Trials Centre University of Sydney Sydney NSW Australia; ^6^ Charles Perkins Centre University of Sydney Sydney NSW Australia; ^7^ Epidemiology and Biostatistics School of Public Health Imperial College London London UK; ^8^ Department of Clinical Pharmacy and Pharmacology University Medical Center Groningen University of Groningen Groningen The Netherlands; ^9^ Kolling Institute Royal North Shore Hospital and University of Sydney Sydney NSW Australia; ^10^ Department of Renal Medicine Royal North Shore Hospital Sydney NSW Australia; ^11^ Stanford Center for Clinical Research Stanford University School of Medicine Stanford CA USA; ^12^ Division of Cardiovascular Medicine Stanford University School of Medicine Stanford CA USA

**Keywords:** canagliflozin, cardiovascular outcomes, magnesium, type 2 diabetes

## Abstract

**Background:**

Patients with type 2 diabetes (T2D) are predisposed to derangements in serum Magnesium (Mg), which may have implications for cardiometabolic events and outcomes. In clinical trials, participants with T2D randomized to sodium‐glucose co‐transporter 2 (SGLT2) inhibitors have shown mild to moderate increases in serum Mg from baseline levels. This post hoc analysis assesses the relation between serum Mg with cardiovascular outcomes in 10,140 participants of the Canagliflozin Cardiovascular Assessment Study (CANVAS) Program.

**Methods:**

We evaluated the association of baseline serum Mg with the primary composite end point of death from cardiovascular causes, non‐fatal myocardial infarction, and non‐fatal stroke, and tested whether this association is modified by baseline serum Mg. Using mediation analysis, we determined whether change in serum Mg post‐randomization mediates the beneficial effect of canagliflozin on cardiovascular outcomes.

**Results:**

Mean serum Mg levels at baseline were 0.77 ± 0.09 mmol/L in both canagliflozin group and placebo groups. The canagliflozin group experienced an average increase in serum Mg by 0.07 mmol/L (95% CI, 0.065–0.072 mmol/L; *p* < .001) for the duration of the trial. We found no association between baseline serum Mg levels and the primary composite end point, and no evidence of effect modification by baseline Mg levels. Change in serum Mg post‐randomization was not a mediator of the effects of canagliflozin on cardiovascular outcomes.

**Conclusions:**

In participants of the CANVAS Program, baseline and post‐randomization serum Mg levels are not associated with cardiovascular outcomes.

## Graphical abstract

In the CANVAS Program, participant baseline serum Mg is not associated with cardiovascular outcomes and does not modify the effect of canagliflozin on cardiovascular outcomes. Canagliflozin‐associated rise in serum Mg levels does not mediate the beneficial effects of canagliflozin on cardiovascular outcomes.

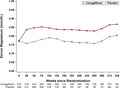

## 1 INTRODUCTION

Magnesium (Mg) is the second most abundant intracellular cation in the body and serves as a cofactor for over 300 enzymatic reactions.^1^ Mg modulates vascular tone and cardiac rhythm and plays an important role in maintaining cardiovascular health.^2^ Overall Mg balance depends on an interplay between intestinal absorption, bone exchange, and faecal and urinary excretion, with the kidney serving as the primary regulator of serum Mg homoeostasis.^2^

Abnormalities in serum Mg levels, especially hypomagnesaemia, have been implicated in various cardiometabolic outcomes.^3^ In mouse models, hypomagnesaemia leads to cardiac and renal inflammation and fibrosis.^4^ In humans, hypomagnesaemia has been associated with atrial fibrillation,^5^ coronary heart disease,^6^ cardiovascular death^6^, ^7^ including sudden cardiac death,^7^, ^8^ and all‐cause mortality.^9^ Patients with type 2 diabetes (T2D) are particularly predisposed to hypomagnesaemia due to poor intestinal absorption and Mg wasting through the kidneys.^10^

Canagliflozin is a sodium‐glucose co‐transporter 2 (SGLT2) inhibitor, which lowers serum glucose by blocking renal glucose reabsorption in the proximal tubule to induce glucosuria. Beyond improvement of glycaemic control and cardiovascular and kidney outcomes, these agents have also been shown to increase serum Mg levels in participants with T2D.^11^, ^12^, ^13^, ^14^, ^15^, ^16^ The Canagliflozin Cardiovascular Assessment Study (CANVAS) Program found that participants randomized to canagliflozin versus placebo showed a 14% risk reduction in major adverse cardiovascular events (MACE), which included cardiovascular death, non‐fatal myocardial infarction and non‐fatal stroke.^12^

This post hoc analysis leverages data from the CANVAS program to determine whether baseline serum Mg levels associates with or modifies the effect of canagliflozin, on cardiovascular outcomes. In addition, given prior data that SGLT2 inhibitors increase serum Mg levels and the association between serum Mg and cardiovascular outcomes, we performed a mediation analysis to assess whether a post‐randomization change in serum Mg mechanistically plays a role in the attenuation of cardiovascular risk with canagliflozin versus placebo.

## 2 METHODS

### 2.1 Study design

The CANVAS Program pooled data from CANVAS and CANVAS‐R trials, which together randomized 10,142 participants, ages ≥30 years with T2D at high risk for cardiovascular events, to canagliflozin versus placebo. Participants in CANVAS were assigned to canagliflozin 100 mg, canagliflozin 300 mg and placebo in a 1:1:1 fashion, while participants in CANVAS‐R were assigned to canagliflozin at an initial dose of 100 mg with option to increase to 300 mg at week 13 (of which 71.4% of participants in the canagliflozin did so), versus placebo. Mean follow‐up time was 188 weeks. Major exclusion criteria included type 1 diabetes, not being on a stable antihyperglycaemic regimen for at least 8 weeks, cardiovascular events within 3 months of screening or planned revascularization, and eGFR <30 ml/min/1.73 m^2^. Further details regarding study design, participant inclusion and exclusion criteria, and main results can be found in the original manuscript.^12^

### 2.2 Exposure: Serum Mg

Serum Mg was collected as part of the Serum Chemistry Panel, and measured at baseline, post‐randomization weeks 6, 13, 18, 26, 39, 52, and then at 26‐week intervals per protocol. Testing was performed by a central laboratory, with exception of local laboratory processing in situations where immediate testing was necessary for clinical care. This analysis includes only participants who had a baseline and post‐baseline Mg measurement up to individual trial completion. We used a SAS^®^ macro to determine whether the exposure should be modelled as continuous/linear, categorical, quadratic or spline. Baseline serum Mg is modelled as a categorical variable, in quintiles, based on optimal model fit and interpretability of the results.^17^

### 2.3 Outcomes

The primary outcome for the present analysis was MACE, a composite of cardiovascular death, non‐fatal myocardial infarction and non‐fatal stroke. Cardiovascular death included death from myocardial infarction, heart failure, stroke, sudden cardiac death, among other related causes. Cardiovascular outcomes were determined by an independent events adjudication committee blinded to the treatment group.

### 2.4 Statistical analyses

The mean change from baseline Mg level by treatment group was determined using a mixed‐model repeated‐measures analysis. A Cox proportional hazards model was used to examine the association of baseline Mg quintile with cardiovascular outcomes, with the third quintile serving as the reference group. Results are reported as hazard ratios (HR) and 95% confidence intervals (CI).

Baseline quintile of serum Mg was assessed as an effect modifier on the effects of canagliflozin on cardiovascular outcomes, to determine whether participants with lower baseline Mg levels benefited differently than participants with normal‐high baseline Mg levels given the expected increase in serum Mg with canagliflozin. A Cox proportional hazards model was used to present the effect of canagliflozin versus placebo on MACE stratified by baseline Mg quintile. This survival model analyses time to first event; any event that occurs any time during the data period is considered an eligible event. Subjects not experiencing an event are censored at the last trial contact date or end of the respective data period, whichever date comes earlier. A multiplicative interaction term was included for canagliflozin and quintile of baseline Mg.

Serum Mg was identified post hoc as a potential mediator, as it is changed by canagliflozin and has been shown to associate with cardiovascular outcomes in prior studies. The counterfactual method was utilized to perform the mediation analysis.^18^

All Cox regression models were stratified by study and history of cardiovascular disease. Covariates in multivariable models included baseline age, sex, race, current smoker, history of hypertension, history of heart failure, duration of diabetes, history of amputation, body mass index (BMI), blood pressure, glycated haemoglobin, cholesterol, level of albuminuria, estimated glomerular filtration rate (eGFR) and diuretic use.

All analyses were completed using SAS Enterprise Guide, version 7.11 (Cary, North Carolina). *P*‐Values < .05 were considered to be statistically significant.

## 3 RESULTS

### 3.1 Baseline characteristics

From the original CANVAS cohort, two participants were missing baseline serum Mg and thus excluded. A total of 10,140 participants were included in the present analysis, with 5794 participants randomized to canagliflozin and 4346 participants randomized to placebo. Mean age of participants at enrolment was 63.3 years, with 35.8% being women (Table 1). In the lower baseline Mg quintiles, there tended to be higher proportions of women, whites, presence of albuminuria, and use of diuretics. Participants with baseline hypomagnesaemia (defined as baseline serum Mg < 0.66 mmol/L) were 9.9%.

**TABLE 1:** Baseline characteristics of the study cohort overall and stratified by quintile of baseline serum Mg

	Total *N* = 10,140	Quintile 1 *N* = 1841	Quintile 2 *N* = 2002	Quintile 3 *N* = 2184	Quintile 4 *N* = 1976	Quintile 5 *N* = 2137
Baseline Mg—mmol/L	0.77 ± 0.09	0.63 ± 0.05	0.72 ± 0.02	0.77 ± 0.01	0.82 ± 0.01	0.89 ± 0.05
Age—year	63.3 ± 8.2	63.8 ± 7.7	62.8 ± 8.3	62.7 ± 8.2	63.2 ± 8.3	64.1 ± 8.5
Female sex—no. (%)	3632 (35.8)	776 (42.2)	783 (39.1)	771 (35.3)	628 (31.8)	674 (31.5)
Race—no. (%)						
White	7942 (78.3)	1568 (85.2)	1628 (81.3)	1725 (79.0)	1499 (75.9)	1522 (71.2)
Asian	1284 (12.7)	124 (6.7)	172 (8.6)	275 (12.6)	305 (15.4)	408 (19.1)
Black	336 (3.3)	54 (2.9)	76 (3.8)	73 (3.3)	63 (3.2)	70 (3.3)
Other	578 (5.7)	95 (5.2)	126 (6.3)	111 (5.1)	109 (5.5)	137 (6.4)
Current smoker—no. (%)	1806 (17.8)	299 (16.2)	375 (18.7)	393 (18.0)	377 (19.1)	362 (16.9)
Duration of diabetes—yr	13.6 (7.8)	14.3 (7.3)	13.7 (7.7)	13.1 (7.5)	13.3 (7.8)	13.5 (8.3)
Disease history—no. (%)						
Hypertension	9123 (90.0)	1716 (93.2)	1833 (91.6)	1962 (89.8)	1734 (87.8)	1878 (87.9)
Heart failure	1460 (14.4)	251 (13.6)	264 (13.2)	327 (15.0)	308 (15.6)	310 (14.5)
Amputation	238 (2.3)	45 (2.4)	45 (2.2)	51 (2.3)	40 (2.0)	57 (2.7)
Atherosclerotic vascular disease						
Coronary	5721 (56.4)	1046 (56.8)	1073 (53.6)	1210 (55.4)	1132 (57.3)	1260 (59.0)
Cerebral	1957 (19.3)	352 (19.1)	374 (18.7)	439 (20.1)	359 (18.2)	433 (20.3)
Peripheral	2113 (20.8)	367 (19.9)	422 (21.1)	456 (20.9)	390 (19.7)	478 (22.4)
Any	7323 (72.2)	1308 (71.0)	1402 (70.0)	1583 (72.5)	1419 (71.8)	1611 (75.4)
Microvascular disease						
Retinopathy	2129 (21.0)	409 (22.2)	423 (21.1)	429 (19.6)	406 (20.5)	462 (21.6)
Nephropathy	1774 (17.5)	360 (19.6)	351 (17.5)	378 (17.3)	339 (17.2)	346 (16.2)
Neuropathy	3110 (30.7)	545 (29.6)	621 (31.0)	682 (31.2)	608 (30.8)	654 (30.6)
Body mass index	32.0 ± 5.9	33.0 ± 6.0	32.5 ± 6.1	32.1 ± 5.8	31.4 ± 5.8	30.9 ± 5.8
Blood pressure—mmHg						
Systolic	136.6 ± 15.8	137.9 ± 15.8	136.9 ± 16.0	136.2 ± 15.5	136.4 ± 15.4	135.9 ± 16.0
Diastolic	77.7 ± 9.7	77.9 ± 9.7	77.9 ± 9.7	78.0 ± 9.5	77.9 ± 9.6	76.9 ± 9.8
Glycated haemoglobin—%	8.2 ± 0.9	8.3 ± 0.9	8.3 ± 0.9	8.3 ± 1.0	8.2 ± 0.9	8.1 ± 0.9
Cholesterol—mmol/L						
Total	4.41.2	4.2 ± 1.1	4.4 ± 1.2	4.4 ± 1.2	4.4 ± 1.1	4.4 ± 1.1
HDL	1.2 ± 0.3	1.2 ± 0.3	1.2 ± 0.3	1.2 ± 0.3	1.2 ± 0.3	1.2 ± 0.3
LDL	2.3 ± 0.9	2.1 ± 0.9	2.3 ± 0.9	2.3 ± 1.0	2.3 ± 0.9	2.4 ± 1.0
Ratio of LDL to HDL	2.0 ± 0.9	1.9 ± 0.9	2.0 ± 0.9	2.1 ± 0.9	2.1 ± 0.9	2.1 ± 0.9
Triglycerides—mmol/L	2.0 ± 1.4	2.2 ± 1.6	2.1 ± 1.6	2.0 ± 1.3	1.9 ± 1.3	1.9 ± 1.2
eGFR—mL/min/1.73 m^2^	76.5 ± 20.5	75.8 ± 19.8	78.9 ± 20.8	78.2 ± 20.1	76.8 ± 20.0	72.7 ± 21.1
Albuminuria—no. (%)						
Median ACR (IQR)—mg/g	12.3 (6.7–42.1)	16.5 (8.0–62.3)	13.2 (7.0–44.6)	11.8 (6.6–37.8)	11.0 (6.1–34.3)	10.5 (6.1–35.4)
Normoalbuminuria	7005 (69.1)	1150 (62.5)	1365 (68.2)	1535 (70.3)	1428 (72.3)	1527 (71.5)
Microalbuminuria	2266 (22.3)	505 (27.4)	464 (23.2)	476 (21.8)	398 (20.1)	423 (19.8)
Macroalbuminuria	760 (7.5)	169 (9.2)	157 (7.8)	151 (6.9)	124 (6.3)	159 (7.4)
Diuretic use—no. (%)	4490 (44.3)	970 (52.7)	893 (44.6)	922 (42.2)	809 (40.9)	896 (41.9)

Plus‐minus values are means ± SD.

Abbreviations: ACR, albumin to creatinine ratio; IQR, interquartile range.

### 3.2 Changes in serum Mg levels during the trial

**FIGURE 1:**
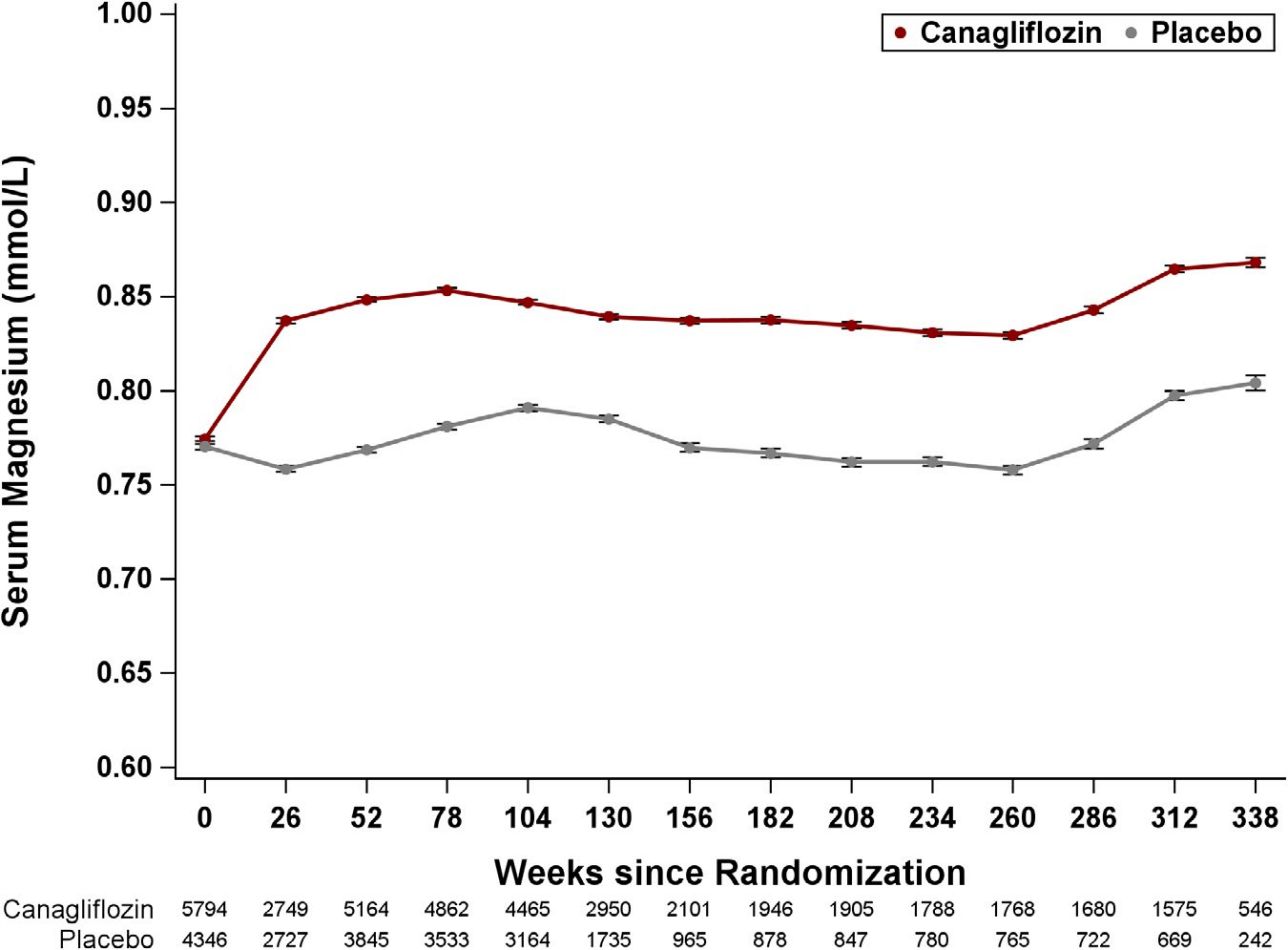
Mean Magnesium values in the canagliflozin and placebo groups over the duration of the trial. Mixed‐model repeated‐measures analysis using all data up to individual trial completion in participants who had a baseline and post‐baseline measurement

Mean baseline serum Mg level was 0.77 ± 0.09 mmol/L in the canagliflozin group and 0.77 ± 0.09 mmol/L in the placebo group. By week 6, the mean serum Mg level increased to 0.86 ± 0.09 mmol/L in the canagliflozin group versus 0.78 ± 0.09 mmol/L in the placebo group. Changes in serum Mg levels from baseline levels were sustained throughout the trial duration (Figure 1). The mean difference in change from baseline serum Mg between the canagliflozin group and the placebo group during the trial was 0.07 mmol/L (95% CI 0.065–0.072 mmol/L, *p* < .001).

### 3.3 Baseline serum Mg and cardiovascular outcomes

**FIGURE 2:**
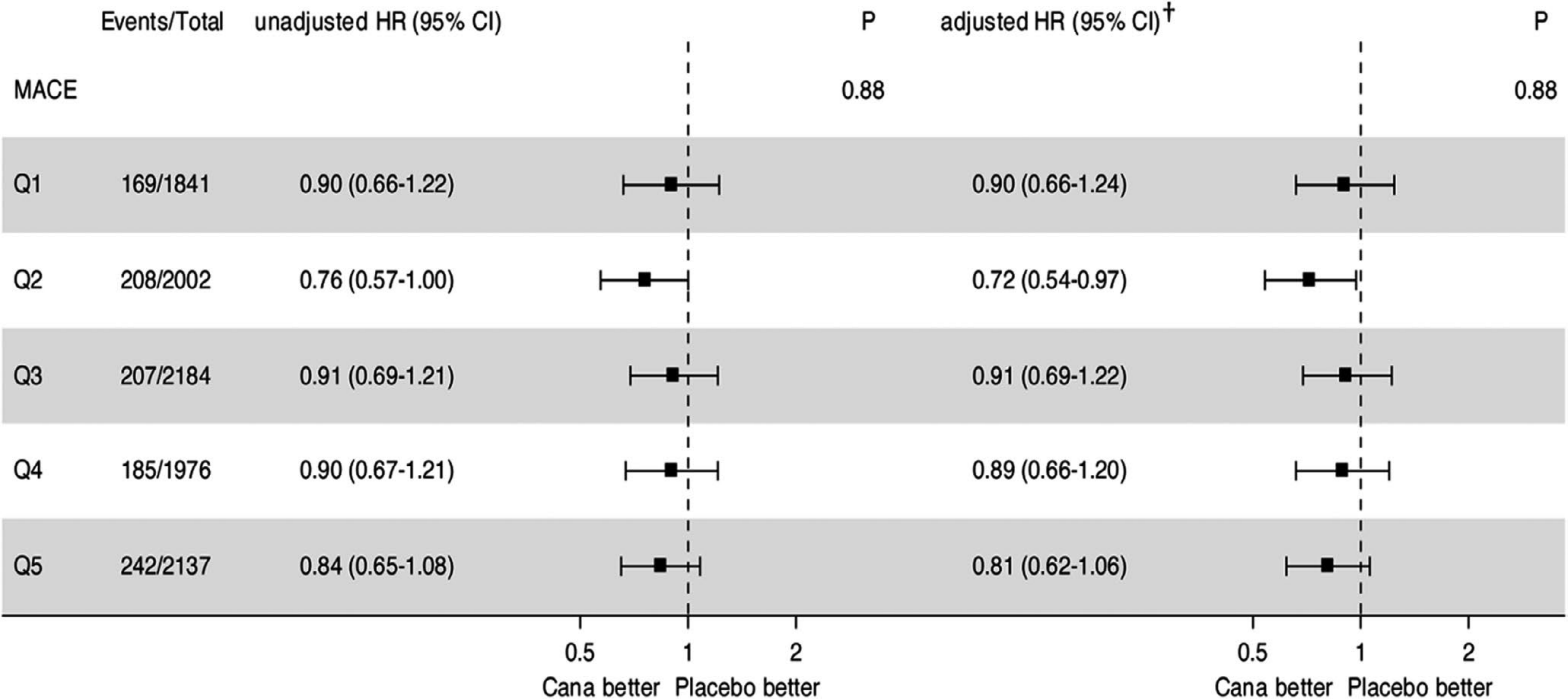
Association between canagliflozin and MACE, and effect modification by baseline serum Mg in unadjusted and adjusted models. Q, quintile. *P*, p‐value for interaction. Models are stratified by study, history of cardiovascular disease, and randomized group. ^†^Adjusted model includes age, sex, race, current smoker, history of hypertension, history of heart failure, duration of diabetes, history of amputation, BMI, systolic BP, glycated haemoglobin, cholesterol, albuminuria (normo, micro, macro), eGFR and diuretic use

We found no significant association between baseline quintile of serum Mg and MACE, cardiovascular death, sudden cardiac death, or heart failure in adjusted models (Table 2). Event rates were low for cardiovascular death, sudden cardiac death and heart failure. Unadjusted hazard ratios between baseline quintile of serum Mg and cardiovascular outcomes can be found in Table S1. We also found no evidence that baseline serum Mg modified the beneficial effect of canagliflozin versus placebo on MACE (Figure 2, *p* = .88).

**TABLE 2:** Association between baseline serum Mg quintile and risk of cardiovascular outcomes in adjusted Cox models

Mg—mmol/L	MACE			Cardiovascular death			Sudden cardiac death			Heart failure		
	Events/total	Adjusted HR (95% CI)	*p*‐Value	Events/total	Adjusted HR (95% CI)	*p*‐Value	Events/Total	Adjusted HR (95% CI)	*p*‐Value	Events/Total	Adjusted HR (95% CI)	*p*‐Value
Q1: 0.63 ± 0.05	169/1841	0.94 (0.76, 1.15)	.20	67/1841	0.92 (0.67, 1.28)	.17	27/1841	0.87 (0.52, 1.46)	.79	53/1841	1.50 (0.98, 2.30)	.32
Q2: 0.72 ± 0.02	208/2002	1.16 (0.95, 1.41)		94/2002	1.34 (0.99, 1.81)		34/2003	1.18 (0.73, 1.91)		51/2002	1.51 (0.99, 2.32)	
Q3:0.77 ± 0.01 (Ref)	207/2184	1.00		85/2184	1.00		36/2184	1.00		37/2184	1.00	
Q4: 0.82 ± 0.01	185/1976	0.93 (0.76, 1.13)		93/1976	1.17 (0.87, 1.57)		33/1976	0.97 (0.60, 1.57)		44/1976	1.23 (0.79, 1.91)	
Q5: 0.89 ± 0.05	242/2137	1.00 (0.83, 1.21)		114/2137	1.10 (0.83, 1.47)		49/2137	1.11 (0.71, 1.73)		58/2137	1.33 (0.87, 2.03)	

Abbreviations: HR, hazard ratio; Q, quintile; Ref, reference.

^a^ Models are stratified by study, history of cardiovascular disease, and randomized group.

^b^ Adjusted models include age, sex, race, current smoker, history of hypertension, history of heart failure, duration of diabetes, history of amputation, BMI, systolic BP, glycated haemoglobin, cholesterol, albuminuria (normo, micro, macro), eGFR and diuretic use.

### 3.4 Change in serum Mg as a mediator

**FIGURE 3:**
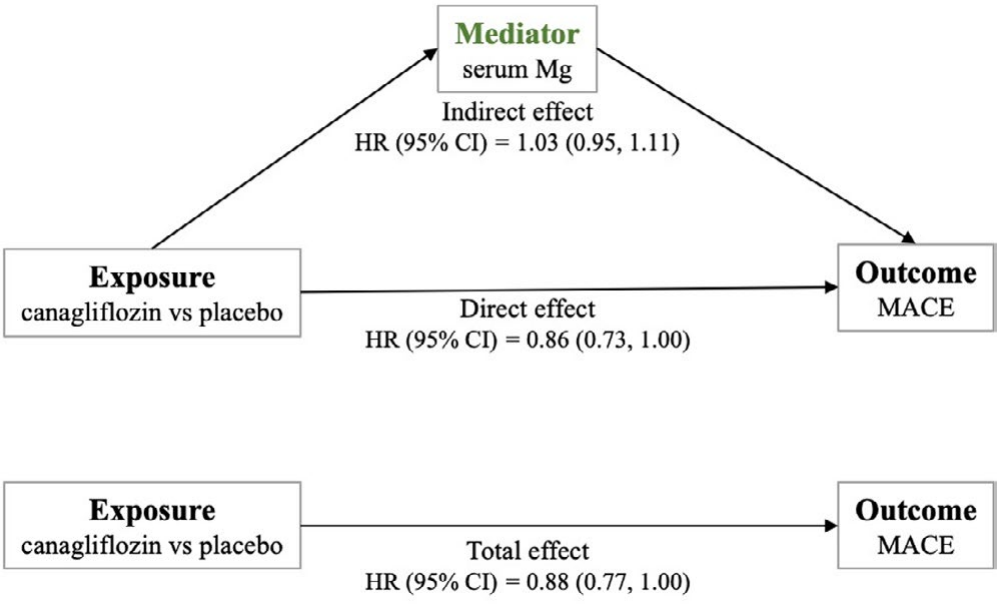
Mediation of effect of canagliflozin on the MACE composite by change in serum Magnesium (Mg). The total effect of canagliflozin on the composite MACE outcome can be deconstructed into the indirect effect mediated by changes in serum Mg and the direct effect, which represents the effect of the exposure through pathways unrelated to serum Mg. Mg magnesium, MACE major adverse cardiovascular events, HR hazard ratio

An exploratory mediation analysis was performed to examine pathways whereby canagliflozin leads to risk reduction in MACE. Mediation analysis seeks to disentangle the total effect of the exposure on the outcome through estimates of the indirect effect (how much of the total intervention effect acts through the mediator), and the direct effect (how much of the total intervention effect is not explained by the mediator). In addition to better understanding of the causal pathway between an exposure and an outcome, identification of mediators may also help identify potential targets for future interventions. The total effect represents the combination of direct effects of canagliflozin on MACE and indirect effects through the intermediate variable, serum Mg, measured post‐randomization (Figure 3).

In this analysis, the direct effect of canagliflozin on MACE was 0.86 (95% CI, 0.73–1.00) and indirect effect through serum Mg was 1.03 (95% CI, 0.95–1.11), with a percent mediation of −18.6% (*p* = .49). Thus, we found no evidence that serum Mg mediates the effect of canagliflozin on MACE. Of note, the discrepancy between the total effect measured from this mediation analysis (HR 0.88; 95% CI, 0.77–1.00) and the original CANVAS analysis (HR 0.86; 95% CI, 0.75–0.97) is due to slight differences in the cohorts included in each analysis. Of the original cohort, our mediation analysis excluded participants missing a baseline serum, follow‐up serum Mg measurements, or if they experienced an event prior to a follow‐up serum Mg measurement.

## 4 DISCUSSION

This post hoc analysis of the CANVAS program included 10,140 participants, allowing for a robust analysis of the treatment effect of canagliflozin on serum Mg concentrations in participants with T2D and elevated cardiovascular risk. Our analysis found that participants randomized to canagliflozin experience sustained increases in serum Mg levels compared to placebo. However, baseline serum Mg in this cohort was not associated with cardiovascular outcomes and did not modify the effect of canagliflozin on cardiovascular outcomes. In addition, we did not find that changes in serum Mg levels mediate the beneficial effects of canagliflozin on cardiovascular outcomes.

The mean difference in serum Mg of 0.07 mmol/L (95% CI, 0.065–0.072 mmol/L) between participants randomized to canagliflozin versus placebo is consistent with prior studies of SGLT2 inhibitors showing mild to moderate increases in serum Mg of 0.05–0.10 mmol/L, supporting evidence of a class effect.^15^, ^19^, ^20^, ^21^ While the mechanism remains unknown, proposed explanations include decreased renal Mg excretion and transcellular shift of serum Mg into intracellular compartments.^22^ Anecdotally, SGLT2 inhibitors have been shown to decrease renal fractional excretion of Mg in patients with T2D and genetic disorders in renal Mg reabsorption. SGLT2 inhibitors induce volume depletion and the renin‐angiotensin system, and angiotensin II may enhance tubular reabsorption of Mg by solvent drag.^23^, ^24^ Other direct or indirect effects on renal Mg transporters may also be possible. SGLT2 inhibitors decrease insulin, which would theoretically shift Mg out of cells. However, a careful euglycaemic hyperinsulinemic clamp study demonstrated only a modest increase in serum Mg.^25^ Thus, larger studies are necessary to elucidate the mechanisms.

Mg is vital in maintenance of calcium, potassium and phosphate homeostasis. While hypomagnesaemia has been inversely associated with risk of atrial and ventricular arrhythmias, coronary artery disease, and cardiovascular death, studies on the association between serum Mg concentrations, dietary Mg intake and cardiovascular outcomes have also yielded conflicting results.^5^, ^6^, ^7^, ^8^, ^9^, ^26^ Rotterdam Study investigators analysed serum Mg samples from 9820 participants and found that those with lower serum Mg had increased risk of coronary artery disease mortality (HR 1.36, 95% CI 1.09 to1.69) and sudden cardiac death (HR 1.54, 95 CI% 1.12–2.11).^7^ The Atherosclerosis Risk in Communities Study recruited 14,232 participants and found that those in the highest compared to lowest quartile of serum Mg had 38% risk reduction in sudden cardiac death (HR 0.62, 95% CI 0.42–0.93). The Paris Prospective II study enrolled 4035 men and found those with serum Mg in the highest category had 40% risk reduction in all‐cause death compared to the lowest category (HR 0.6, 95% 0.4–0.8), although there was no association for cardiovascular death (HR 0.6, 95% CI 0.2–1.2).^27^ Meanwhile, data from the Framingham Heart Study offspring cohort showed no association between the highest quartile of serum Mg and incidence of cardiovascular disease (HR 0.91, 95% CI 0.72–1.17, *p* = .47) or all‐cause mortality (0.92, 95% CI 0.68–1.24, *p* = .57).^28^ These conflicting results are likely due to differences in study population, methodology and outcomes of interest. The majority of prior studies have been observational and thus limited in their ability in establishing a causal pathway between serum Mg and cardiovascular disease and/or death. Observational studies of dietary Mg intake have shown inverse association with cardiovascular outcomes, but these are subject to recall bias or other confounding as they often rely on data collection through self‐administered questionnaires.^29^, ^30^

Our study did not find an association between serum Mg levels and cardiovascular outcomes; however, it also differs significantly from prior observational studies in terms of patient cohort, study design and follow‐up. All participants randomized in the CANVAS Program had long‐standing T2D, 65.6% had history of cardiovascular disease, and 30.3% had micro‐macroalbuminuria. These comorbidities contrast from studies that recruited younger, healthier participants with no underlying cardiovascular disease to study incident cardiovascular outcomes. In addition, the median follow‐up in CANVAS was 2.4 years, whereas follow‐up time in other studies spanned up to 19 years, allowing for time to accrue greater numbers of events.^9^ Strengths of the CANVAS study include outcomes adjudicated by a committee, while some prior studies relied on less definitive sources such as death certificates, leaving risk for misclassification.^9^ The CANVAS Program also had the benefit of access to repeated Mg measurements whereas other analyses were based on a single baseline serum Mg level.^7^ Finally, prior randomized controlled trials studying the effect of Mg supplementation in various cardiovascular outcomes have been limited in number, with results being equivocal. Three larger trials studying intravenous Mg supplementation in suspected acute myocardial infarction showed inconsistent results in mortality benefit, and a meta‐analysis including these and other trials found it unlikely that Mg reduces mortality in acute myocardial infarction but may reduce incidence of ventricular and other severe arrhythmias.^30^, ^31^, ^32^ There remains a paucity of well‐designed trials examining the potential causal role of Mg and cardiovascular outcomes, including disease incidence and death.

Improved cardiovascular outcomes with canagliflozin do not appear to be mediated by the change in serum Mg with canagliflozin. There was no evidence that the canagliflozin‐induced rise in serum Mg mediated the beneficial effects of canagliflozin on cardiovascular outcomes. In a meta‐analysis of prospective studies with moderate inter‐study heterogeneity (*I* ^2^ = 49.5%), a 0.2 mmol/L incremental change in circulating Mg was associated with a 30% risk reduction in cardiovascular disease, including incidence and death (RR 0.7, 95% CI 0.56–0.88). Of the nine studies, five were conducted in the United States, the other four conducted in Europe; eight studies had cohorts with ≤10% diabetes; four excluded participants with baseline cardiovascular disease and no studies reported prevalence of chronic kidney disease. There was no association with circulating Mg and ischaemic heart disease or mortality.^33^ A possible explanation for differences in our study results may be that physiologically, the mean difference of 0.07 mmol/L between the canagliflozin and placebo groups may not have been large enough to warrant a clinical effect. Alternatively, other direct and indirect effects of SGLT2 inhibitors may be responsible for their cardiovascular benefits. In a mediation analysis of empagliflozin using data from the EMPA‐REG trial, the most significant mediators for cardiovascular death were changes in haemoglobin and haematocrit, possibly reflecting a reduction in circulatory load.^34^ A mediation analysis using data from the CANVAS Program also found that markers of plasma volume, in addition to serum urate and urine albumin‐to‐creatinine ratio, had the largest mediation effects in canagliflozin versus placebo and reduction of heart failure. Serum Mg levels were not investigated as potential mediators in that analysis. Furthermore, while Mg supplementation has been studied in small T2D cohorts and appears to improve glycaemic control and insulin sensitivity, there is lack of clinical evidence that Mg supplementation in patients with T2D experience improves cardiovascular health.^35^, ^36^

An inherent limitation of any post hoc analysis includes the possibility that associations found in the study may be spurious; all analyses are exploratory and require further clinical investigation. We also note limitations specific to this study. Due to incomplete medication information for all participants, there was inability to adjust for specific agents that may decrease serum Mg (eg thiazide‐type diuretics, proton pump inhibitors) or medications that may increase serum Mg (eg mineralocorticoid antagonists, amiloride). There was no information on participants’ dietary Mg intake or alcohol use, the latter of which is associated with Mg depletion due to low intake, low body stores and renal wasting.^37^, ^38^ We also did not have information on urinary levels of Mg to determine whether participants experienced changes in fractional excretion of Mg. A final consideration is that serum Mg may not be the most accurate measurement of total body stores. Over 99% of total body Mg is stored in bone, muscles and soft tissue, with <1% contained in the serum and red blood cells.^2^ Normal serum Mg levels may be seen in patients with Mg deficiency, typically due to recruitment of intracellular stores (rapidly through muscle stores, and over weeks through bone stores).^39^, ^40^, ^41^ However, serum Mg measurements remain the most commonly used test by clinicians due to relative ease and cost.^41^

## 5 CONCLUSION

In the CANVAS Program, participants randomized to canagliflozin showed a mean increase in serum Mg levels during the course of the study; however, there was no association between baseline serum Mg levels and cardiovascular outcomes, and the beneficial effect of canagliflozin does not vary by baseline serum Mg. Changes from baseline serum Mg levels in the canagliflozin group during the trial did not mediate the beneficial effects of canagliflozin on cardiovascular outcomes. Given that this investigation is exploratory, further physiologic or mechanistic studies specifically designed to elucidate the relationship between serum Mg and cardiovascular outcomes may be beneficial.

## CONFLICT OF INTEREST

Katherine M. Wang and JingWei Li—None. Vivek Bhalla reports ownership stock in Pyrames, consultant fees from Maxim Integrated, service on scientific advisory boards for Relypsa, and is a Co‐Site Investigator for the CALM‐2 trial, sponsored by Vascular Dynamics, Inc., outside the submitted work. Meg J. Jardine is responsible for research projects that have received unrestricted funding from Gambro, Baxter, CSL, Amgen, Eli Lilly, and Merck Sharpe Dohme; serves on Steering Committees sponsored by CSL and Janssen, has served on advisory boards sponsored by Akebia, AstraZeneca, Baxter, Boehringer Ingelheim, and Vifor; and has spoken at scientific meetings sponsored by Amgen, Vifor and Janssen; with any consultancy, honoraria, or travel support paid to her institution. Bruce Neal has served on Steering Committees for Janssen; and has served on advisory boards and as a consultant for Janssen, Mitsubishi Tanabe Pharma Corporation, Merk Sharpe Dohme, and Servier, with all fees paid to his institution. Dick de Zeeuw reports serving on advisory boards and/or as a speaker for Bayer, Boehringer Ingelheim, Fresenius, Mundipharma, Mitsubishi Tanabe; serving on Steering Committees and/or as a speaker for AbbVie and Janssen; and serving on Data Safety and Monitoring Committees for Bayer. Greg Fulcher has received honoraria from Janssen, Novo Nordisk, MSD and Boehringer and research support from Novo Nordisk. Vlado Perkovic has received fees for Advisory Boards, Steering Committee roles, or Scientific Presentations from Abbvie, Amgen, Astellas, Astra Zeneca, Bayer, Baxter, BMS, Boehringer Ingelheim, Chinnook, Dimerix, Durect, Eli Lilly, Gilead, GSK, Janssen, Merck, Metavant, Mitsubishi Tanabe, Mundipharma, Novartis, Novo Nordisk, Pfizer, Pharmalink, Relypsa, Retrophin, Sanofi, Servier, Vifor, Vitae, Uptodate and Tricida. Kenneth W. Mahaffey's financial disclosures can be viewed at http://med.stanford.edu/profiles/kenneth‐mahaffey. Tara I. Chang reports serving as a consultant for Novo Nordisk, Tricida, Gilead, and Bayer unrelated to the submitted work; received support from Janssen and served as a U.S. national leader and events adjudication committee member for CREDENCE; and served on an advisory board sponsored by AstraZeneca.

## AUTHOR CONTRIBUTION

KMW, JL, VB, TIC conceived and designed the study. JL analysed the data. KMW, VB, and TIC drafted the manuscript. All authors critically reviewed the manuscript and provided final approval of the version to be published.

## Supporting information

Table S1

## Data Availability

Data from the CANVAS Program is available in the public domain via the Yale University Open Data Access Project (YODA; http://yoda.yale.edu/).

## References

[1] Saris NEL , Mervaala E , Karppanen H , Khawaja JA , Lewenstam A . Magnesium ‐ An update on physiological, clinical and analytical aspects. Clin Chim Acta. 2000;294(1‐2):1‐26. 10727669 10.1016/s0009-8981(99)00258-2

[2] Jahnen‐Dechent W , Ketteler M . Magnesium basics. Clin Kidney J. 2012;5:3‐14. 10.1093/ndtplus/sfr163 PMC4455825 26069819

[3] Geiger H , Wanner C . Magnesium in disease. Clin Kidney J. 2012;5:25‐38. 10.1093/ndtplus/sfr165 PMC4455821 26069818

[4] Rios FJ , Zou Z‐G , Harvey AP , et al. Chanzyme TRPM7 protects against cardiovascular inflammation and fibrosis. Cardiovascular Research. 2020;116(3):721–735. 10.1093/cvr/cvz164 31250885 PMC7252442

[5] Khan AM , Lubitz SA , Sullivan LM , et al. Low serum magnesium and the development of atrial fibrillation in the community: the Framingham Heart Study. Circulation. 2013;127(1):33‐38. 23172839 10.1161/CIRCULATIONAHA.111.082511 PMC3541701

[6] Gartside PS , Glueck CJ . The important role of modifiable dietary‐characteristics and behavioral‐characteristics in the causation and prevention of coronary‐heart‐disease hospitalization and mortality ‐ the prospective NHANES‐I follow‐up‐study. J Am Coll Nutr. 1995;14(1):71‐79. 7706615 10.1080/07315724.1995.10718476

[7] Kieboom BCT , Niemeijer MN , Leening MJG , et al. Serum Magnesium and the Risk of Death From Coronary Heart Disease and Sudden Cardiac Death. Journal of the American Heart Association. 2016;5(1). 10.1161/jaha.115.002707 PMC4859391 26802105

[8] Misialek JR , Lopez FL , Lutsey PL , et al. Serum and dietary magnesium and incidence of atrial fibrillation in Whites and in African Americans ‐ Atherosclerosis Risk in Communities (ARIC) Study. Circ J. 2013;77(2):323‐329. 23047297 10.1253/circj.cj-12-0886 PMC4228988

[9] Ford ES . Serum magnesium and ischaemic heart disease: findings from a national sample of US adults. Int J Epidemiol. 1999;28(4):645‐651. 10480691 10.1093/ije/28.4.645

[10] Kurstjens S , de Baaij JHF , Bouras H , Bindels RJM , Tack CJJ , Hoenderop JGJ . Determinants of hypomagnesemia in patients with type 2 diabetes mellitus. Eur J Endocrinol. 2017;176(1):11‐19. 27707767 10.1530/EJE-16-0517

[11] Perkovic V , de Zeeuw D , Mahaffey KW , et al. Canagliflozin and renal outcomes in type 2 diabetes: results from the CANVAS Program randomised clinical trials. Lancet Diabetes Endocrinol. 2018;6(9):691‐704. 29937267 10.1016/S2213-8587(18)30141-4

[12] Neal B , Perkovic V , Mahaffey KW , et al. Canagliflozin and cardiovascular and renal events in type 2 diabetes. N Engl J Med. 2017;377(7):644‐657. 28605608 10.1056/NEJMoa1611925

[13] Zinman B , Wanner C , Lachin JM , et al. Empagliflozin, cardiovascular outcomes, and mortality in type 2 diabetes. N Engl J Med. 2015;373(22):2117‐2128. 26378978 10.1056/NEJMoa1504720

[14] Perkovic V , Jardine MJ , Neal B , et al. Canagliflozin and Renal Outcomes in Type 2 Diabetes and Nephropathy. New England Journal of Medicine. 2019;380(24):2295–2306. 10.1056/nejmoa1811744 30990260

[15] Tang HL , Zhang X , Zhang JJ , et al. Elevated serum magnesium associated with SGLT2 inhibitor use in type 2 diabetes patients: a meta‐analysis of randomised controlled trials. Diabetologia. 2016;59(12):2546‐2551. 27628105 10.1007/s00125-016-4101-6

[16] List JF , Woo V , Morales E , Tang W , Fiedorek FT . Sodium‐glucose cotransport inhibition with dapagliflozin in type 2 diabetes. Diabetes Care. 2009;32(4):650‐657. 19114612 10.2337/dc08-1863 PMC2660449

[17] Liu S , Stedman MR . A SAS^®^ macro for covariate specification in linear, logistic and survival regression " Paper 1223‐2017. Proceedings of the SAS Global 2017 Conference, Orlando FL. Available at http://support.sas.com/resources/papers/proceedings17/1223‐2017.pdf.

[18] Li JW , Woodward M , Perkovic V , et al. Mediators of the effects of canagliflozin on heart failure in patients with type 2 diabetes. Jacc‐Heart Failure. 2020;8(1):57‐66. 31676303 10.1016/j.jchf.2019.08.004

[19] Tang HL , Fang ZW , Wang TS , Cui W , Zhai S , Song YQ . Meta‐analysis of effects of sodium‐glucose cotransporter 2 inhibitors on cardiovascular outcomes and all‐cause mortality among patients with type 2 diabetes mellitus. Am J Cardiol. 2016;118(11):1774‐1780. 27666177 10.1016/j.amjcard.2016.08.061

[20] Gilbert RE , Mende C , Vijapurkar U , Sha S , Davies MJ , Desai M . Effects of canagliflozin on serum magnesium in patients with type 2 diabetes mellitus: a post hoc analysis of randomized controlled trials. Diabetes Therapy. 2017;8(2):451‐458. 28197834 10.1007/s13300-017-0232-0 PMC5380494

[21] Toto RD , Goldenberg R , Chertow GM , et al. Correction of hypomagnesemia by dapagliflozin in patients with type 2 diabetes: A post hoc analysis of 10 randomized, placebo‐controlled trials. Journal of Diabetes and its Complications. 2019;33(10):107402. 10.1016/j.jdiacomp.2019.06.007 31375422

[22] Ray EC , Boyd‐Shiwarski CR , Liu P , Novacic D , Cassiman D . SGLT2 inhibitors for treatment of refractory hypomagnesemia: a case report of 3 patients. Kidney Med 2020;2:359‐364. 32734255 10.1016/j.xkme.2020.01.010 PMC7380441

[23] Ohara K , Masuda T , Murakami T , et al. Effects of the sodium‐glucose cotransporter 2 inhibitor dapagliflozin on fluid distribution: A comparison study with furosemide and tolvaptan. Nephrology. 2019;24(9):904‐911. 30578654 10.1111/nep.13552

[24] Schork A , Saynisch J , Vosseler A , et al. Effect of SGLT2 inhibitors on body composition, fluid status and renin–angiotensin–aldosterone system in type 2 diabetes: a prospective study using bioimpedance spectroscopy. Cardiovascular Diabetology. 2019;18(1). 10.1186/s12933-019-0852-y PMC6451223 30953516

[25] Xu LHR , Maalouf NM . Effect of acute hyperinsulinemia on magnesium homeostasis in humans. Diabetes/Metabolism Research and Reviews. 2017;33(2):e2844. 10.1002/dmrr.2844 27546733

[26] Al Alawi AM , Majoni SW , Falhammar H . Magnesium and Human Health: Perspectives and Research Directions. International Journal of Endocrinology. 2018;2018:1–17. 10.1155/2018/9041694 PMC5926493 29849626

[27] Leone N , Courbon D , Ducimetiere P , Zureik M . Zinc, copper, and magnesium and risks for all‐cause, cancer, and cardiovascular mortality. Epidemiology. 2006;17(3):308‐314. 16570028 10.1097/01.ede.0000209454.41466.b7

[28] Khan AM , Sullivan L , McCabe E , Levy D , Vasan RS , Wang TJ . Lack of association between serum magnesium and the risks of hypertension and cardiovascular disease. Am Heart J. 2010;160(4):715‐720. 20934566 10.1016/j.ahj.2010.06.036 PMC2953800

[29] Kokubo Y , Saito I , Iso H , et al. Dietary magnesium intake and risk of incident coronary heart disease in men: A prospective cohort study. Clin Nutr. 2018;37(5):1602‐1608. 28843443 10.1016/j.clnu.2017.08.006

[30] Abbott RD , Ando F , Masaki KH , et al. Dietary magnesium intake and the future risk of coronary heart disease (The Honolulu Heart Program). Am J Cardiol. 2003;92(6):665‐669. 12972103 10.1016/s0002-9149(03)00819-1

[31] Collins R , Peto R , Flather M , et al. ISIS‐4 ‐ a randomized factorial trial assessing early oral captopril, oral mononitrate, and intravenous magnesium‐sulfate in 58,050 patients with suspected acute myocardial‐infarction. Lancet. 1995;345(8951):669‐685. 7661937

[32] Woods KL , Fletcher S . Long‐term outcome after intravenous magnesium‐sulfate in suspected acute myocardial‐infarction ‐ the 2nd leicester intravenous magnesium intervention trial (LIMIT‐2). Lancet. 1994;343(8901):816‐819. 7908076 10.1016/s0140-6736(94)92024-9

[33] Del Gobbo LC , Imamura F , Wu JHY , Otto MCD , Chiuve SE , Mozaffarian D . Circulating and dietary magnesium and risk of cardiovascular disease: a systematic review and meta‐analysis of prospective studies. Am J Clin Nutr. 2013;98(1):160‐173. 23719551 10.3945/ajcn.112.053132 PMC3683817

[34] Inzucchi SE , Zinman B , Fitchett D , et al. How does empagliflozin reduce cardiovascular mortality? Insights from a mediation analysis of the EMPA‐REG OUTCOME Trial. Diabetes Care. 2018;41(2):356‐363. 29203583 10.2337/dc17-1096

[35] Courivaud C , Davenport A . Magnesium and the risk of all‐cause and cardiac mortality in hemodialysis patients: agent provocateur or innocent bystander? Kidney Int. 2014;85(1):17‐20. 24380904 10.1038/ki.2013.301

[36] Lima MDL , Cruz T , Pousada JC , Rodrigues LE , Barbosa K , Cangucu V . The effect of magnesium supplementation in increasing doses on the control of type 2 diabetes. Diabetes Care. 1998;21(5):682‐686. 9589224 10.2337/diacare.21.5.682

[37] Kalbfleisch JM , Ginn HE , Smith WO , Lindeman RD . Effects of ethanol administration on urinary excretion of magnesium and other electrolytes in alcoholic and normal subjects. J Clin Invest. 1963;42(9):1471. 14060991 10.1172/JCI104831 PMC289421

[38] Demarchi S , Cecchin E , Basile A , Bertotti A , Nardini R , Bartoli E . Renal tubular dysfunction in chronic alcohol‐abuse ‐ effects of abstinence. N Engl J Med. 1993;329(26):1927‐1934. 8247056 10.1056/NEJM199312233292605

[39] Ismail Y , Ismail AA , Ismail AAA . The underestimated problem of using serum magnesium measurements to exclude magnesium deficiency in adults; a health warning is needed for "normal" results. Clin Chem Lab Med. 2010;48(3):323‐327. 20170394 10.1515/CCLM.2010.077

[40] Costello RB , Elin RJ , Rosanoff A , et al. Perspective: the case for an evidence‐based reference interval for serum magnesium: the time has come. Adv Nutr. 2016;7(6):977‐993. 28140318 10.3945/an.116.012765 PMC5105038

[41] DiNicolantonio JJ , O'Keefe JH , Wilson W . Subclinical magnesium deficiency: a principal driver of cardiovascular disease and a public health crisis. Open. Heart. 2018;5(1):e000668. 10.1136/openhrt-2017-000668 PMC5786912 29387426

