# Supplementary material for: Canagliflozin, serum magnesium and cardiovascular outcomes—Analysis from the CANVAS Program
Source: Endocrinol Diabetes Metab. 2021 Mar 13;4(3):e00247. doi: 10.1002/edm2.247 (PMC8279612; doi:10.1002/edm2.247)
Supplement: Supplementary file 1 — Table S1 [file EDM2-4-e00247-s001.docx]

**Supplementary Table 1:** **Unadjusted hazard ratios between baseline serum Mg quintile and risk of cardiovascular outcomes**

|  | MACE | | Cardiovascular Death | | Sudden Cardiac Death | | Heart Failure | |
| --- | --- | --- | --- | --- | --- | --- | --- | --- |
|  | Unadjusted HR (95% CI) | P-value | Unadjusted HR (95% CI) | P-value | Unadjusted HR (95% CI) | P-value | Unadjusted HR (95% CI) | P-value |
| Mg – mmol/L |  | 0.18 |  | 0.23 |  | 0.72 |  | 0.08 |
| Q1: 0.63±0.05 | 1.01 (0.82, 1.24) |  | 0.99 (0.72, 1.37) |  | 0.95 (0.58, 1.57) |  | 1.77 (1.16, 2.70) |  |
| Q2: 0.72±0.02 | 1.18 (0.98, 1.43) |  | 1.34 (1.00, 1.79) |  | 1.14 (0.71, 1.82) |  | 1.58 (1.04, 2.42) |  |
| Q3: 0.77±0.01 (Ref) | 1.00 |  | 1.00 |  | 1.00 |  | 1.00 |  |
| Q4: 0.82±0.01 | 0.94 (0.77, 1.14) |  | 1.15 (0.86, 1.55) |  | 0.96 (0.60, 1.54) |  | 1.27 (0.82, 1.97) |  |
| Q5: 0.89±0.05 | 1.08 (0.90, 1.30) |  | 1.23 (0.93, 1.63) |  | 1.24 (0.81, 1.91) |  | 1.51 (1.00, 2.27) |  |
